# Assessing the adequacy of lymph node yield for different tumor stages of colon cancer by nodal staging scores

**DOI:** 10.1186/s12885-017-3491-2

**Published:** 2017-07-25

**Authors:** Zhenyu Wu, Guoyou Qin, Naiqing Zhao, Huixun Jia, Xueying Zheng

**Affiliations:** 1 Department of Biostatistics and Key Laboratory of Public Health Safety, School of Public Health, Fudan University, Shanghai, 200032 China; 2 Collaborative Innovation Center of Social Risks Governance in Health, Fudan University, 130 Dongan Road, Shanghai, 200032 China; 3 Center for Biomedical Statistics, Fudan University Shanghai Cancer Center, Shanghai, 200032 China

**Keywords:** Colon cancer, False-negative rate, Lymph node, Tumor stage

## Abstract

**Background:**

According to the current official guidelines, at least 12 lymph nodes (LNs) are qualified as an adequate sampling for colon cancer patients. However, patients evaluated with less nodes were still common in the United States, and the prevalence of positive nodal disease may be under-estimated because of the false-negative assessment. In this study, we present a statistical model that allows preoperative determination of the minimum number of lymph nodes needed to confirm a node-negative disease with certain confidence.

**Methods:**

Adenocarcinoma colon cancer patients with stage T1-T3, diagnosed between 2004 and 2013, who did not receive neoadjuvant therapies and had at least one lymph node pathologically examined, were extracted from the Surveillance, Epidemiology and End Results (SEER) database. A beta binomial distribution was used to estimate the probability of an occult nodal disease is truly node-negative as a function of total number of LNs examined and T stage.

**Results:**

A total of 125,306 patients met study criteria; and 47,788 of those were node-positive. The probability of falsely identifying a patient as node-negative decreased with an increasing number of nodes examined for each stage, and was estimated to be 72% for T1 and T2 patients with a single node examined and 57% for T3 patients with a single node examined. To confirm an occult nodal disease with 90% confidence, 3, 8, and 24 nodes need to be examined for patients from stage T1, T2, and T3, respectively.

**Conclusions:**

The false-negative rate of diagnosed node negative, together with the minimum number of examined nodes for adequate staging, depend preoperatively on the clinical T stage. Predictive tools can recommend a threshold on the minimum number of examined nodes regarding to the favored level of confidence for each T stage.

**Electronic supplementary material:**

The online version of this article (doi:10.1186/s12885-017-3491-2) contains supplementary material, which is available to authorized users.

## Background

Colon cancer is the most common digestive system malignant tumor, accounting for approximately one thirds of the estimated new cases, in the United States in 2016 [1]. Although the incidence rate of colon cancer declines dramatically, decreased by more than 4% per year in both men and women from 2008 to 2012 [2], it is estimated that 95,270 cases were newly developed in 2016 [1]. Given the fact that about 49,000 Americans died of this disease in 2016 [1], improving the medical and clinical care of colon cancer remains a great challenge. Accurate evaluation of loco-regional lymph nodes (LNs) status is essential for assessing the stage of disease, planning the effective systematic therapies, and predicting the prognosis of these patients [3–8]. Therefore, the detection of positive LNs is critical and a great deal of efforts have been made on determination of the threshold of LNs need to be retrieved. Apparently, if there was too few LNs examined during the surgery, there would be a great chance of being “under-staging” or falsely identifying a node-positive patient as node-negative. Recommendations on lymph node sampling varied from 6 to 21 [9–14], however, most of the studies have suggested that an examination of at least 12 regional lymph nodes is reasonable for nodal evaluation for colon cancer patients [15–18]. Official guidelines, such as those announced by the American Joint Committee on Cancer, the American Society of Clinical Oncology, American College of Surgeons, the National Quality Forum, and the National Comprehensive Cancer Network also accepted a minimum of 12 LNs as a standard retrieved from a patient with colon cancer [19–21].

Despite these guidelines, false-negative nodal staging caused by inadequacy of lymph node retrieval exists on a broad scale. Previous studies showed certain interests in developing tools which can help physicians and pathologists predict the probability of missing nodal disease [12, 22]. In the context of tumor-node-metastasis staging, T stage was considered as the only stratified covariate in those tools. However, some other key factors, such as therapies and characteristics of patients, were not involved. Patients received neoadjuvant therapy had significantly fewer nodes assessed than patients who underwent surgery alone [23]. The aims of this study were to present a new statistical model to calculate the false-negative probability of occult nodal disease as a function of the number of examined LNs and the T stage, using the first primary colon patients without neoadjuvant therapy from a nationwide database. A larger value of the improved nodal staging score (NSS) indicates greater certainty on the node-negative status of a patient.

## Methods

### Data source

Data for the current study were extracted from the Surveillance, Epidemiology, and End Results (SEER)-Medicare linked database. The SEER program of National Cancer Institute collects demographics, tumor characteristics, and survival data from 17 population-based cancer registries throughout the United States, covering approximately 28% of the US population [24]. The SEER-Medicare database has been described in detail elsewhere [25].

### Patients

**Fig. 1:**
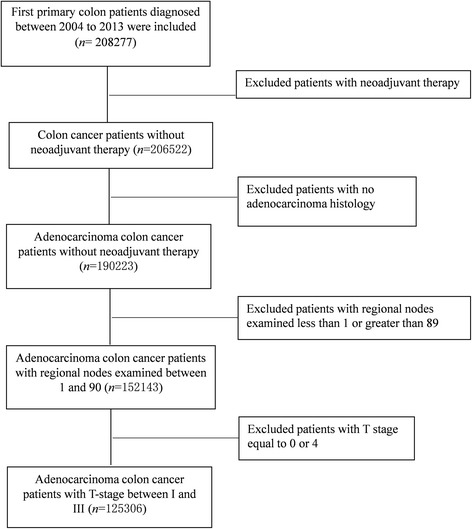
Flow diagram of colon cancer patients enrolled from the Surveillance, Epidemiology, and End Results (SEER)-Medicare linked database

Only first primary (i.e., only primary cancer or first of two or more primary cancers) colon cancer patients diagnosed between 2004 and 2013 were included. Patients were excluded if they 1) have been treated with neoadjuvant therapy; 2) have histology type other than adenocarcinoma; 3) have no lymph node examined or the number of lymph nodes examined was not available; 4) T stage equals 0 or 4. A study flowchart is presented in Fig. 1.

### Statistical analysis

The probability that a node-negative patient has nodal disease can be computed using the following algorithm:

1. Compute the probability of missing a positive node as a function of the number of examined nodes, which depends on the number of examined nodes and on T stage.
2. Compute the corrected prevalence of nodal disease as a function of T stage, using the probability of missing a positive node.
3. Compute the NSS. This is the probability that a pathologically node-negative patient is actually free of nodal disease, which is calculated from the prevalence and the probability of missing a positive node.

### Probability of missing a positive node

We adapted a beta binomial distribution to estimate the probability of missing a positive node as a function of total number of examined nodes, only using node-positive patients. Two key assumptions underlie this step: (1) There are no false-positives, and (2) sensitivity is the same for node-positive and node-negative patients. The probability of false-negative depends on the number of examined nodes and on T stage:

$$ P\left({FN}_{m,T}\ \right)=\frac{Beta\left({\alpha}_T,{\beta}_T+m\right)}{Beta\left({\alpha}_T,{\beta}_T\right)}, $$

where *m* denotes the number of nodes examined from 1 to 89, *T* denotes the stage of tumor from T1-T3, and *Beta*() represents the beta function. For each tumor stage, *α*_*T*_ and *β*_*T*_ are parameters that characterize the underlying intensity of nodal disease to be estimated from the individual patient data using maximum likelihood approach via VGAM package in R version 3.2.4.

### Estimation of prevalence of nodal disease

The observed prevalence (OP) is an underestimate and needed to be adjusted for false negatives. This was done in two steps. The first step estimates the number of false negative #*FN*_*m* , *T*_ as a function of number of examined nodes (*m*) and stage (*T*):

$$ \#{FN}_{m,T}=\frac{P{\left({FN}_{m,T}\ \right)}^{\ast }\ \left(\#{TP}_{m,T}\right)}{1-P\left({FN}_{m,T}\ \right)}, $$

where #*TP*_*m* , *T*_ is the number of true positives for a given number of examined nodes (*m*) and stage (*T*). The second step obtains the corrected prevalence (CP) for each stage by summing over all the number of examined nodes (*m*):

$$ {CP}_T=\frac{\sum_m\left(\#{TP}_{m,T}+\#{FN}_{m,T}\right)}{\sum_m\left(\#{TP}_{m,T}+\#{TN}_{m,T}+\#{FN}_{m,T}\right)}=\frac{\sum_m\left(\#{TP}_{m,T}+\#{FN}_{m,T}\right)}{All\  Patients}. $$

### Nodal staging score

We assessed adequate staging by computing the NSS, the probability that a pathologically LN-negative patient is indeed free of nodal metastasis:

$$ {NSS}_{m,T}=\frac{1-{CP}_T}{1-{CP}_T+{CP_T}^{\ast }P\left({FN}_{m,T}\ \right)}. $$

### Confidence intervals

Precision of the reported estimates was assessed by creating 1000 bootstrap samples from the entire data set and replicating the estimation process. The 2.5th and 97.5th percentiles were used as the lower and upper 95% confidence intervals for the corresponding estimates, respectively.

## Results

A total of 125,306 qualified patients were involved in our analyses. The proportions of patients with stage T1, T2 and T3 primary tumor were 14.51%, 17.04% and 68.45%, respectively. The median number of LNs was gradually increased with T stage, from 13 to 16. In addition, the proportion of ≥12 LNs examined and the rate of node-positivity were compared. Most of the enrolled patients were examined with more than 12 nodes, however, the highest node-positive rate was observed in patients with T3 stage. As expected, the rate of patients with positive node was lowest in T1 stage (11.12%) and highest in T3 stage (48.54%). The detailed summaries of patients and LNs were shown in Table 1.

**Table 1:** Descriptions of enrolled patients and lymph nodes examined

T stage	*N*(%)	LNE(*M*)	Rate of ≥12 LNE(%)	Proportion of node-positivity (%)
T1	18,181(14.51)	13	60.26	11.12
T2	21,355(17.04)	15	72.55	19.35
T3	85,770(68.45)	16	77.92	48.54

*LNE* lymph nodes examined, *M* median

The distribution of the percentage of positive metastatic LNs among all patients with at least one positive node (*n* = 47,788) was fit using a beta-binomial distribution with resulting model parameter estimates of *α* = 1.130 (95% CI, 1.111 to 1.149) and *β* = 3.201 (95% CI, 3.128 to 3.288) (Table 2). Stratified by tumor stages (T stage), the resulting parameters were *α*_1_= *α*_2_= 1.960 (95% CI, 1.813 to 2.119) and *β*_1_= *β*_2_=10.453 (95% CI, 9.335 to 11.549) for stages T1 and T2 (estimated by *n* = 6153 patients in stage T1 and T2 with at least one positive node). For stage T3, *α*_3_=1.117 (95% CI, 1.100 to 1.136) and *β*_3_=2.957 (95% CI, 2.886 to 3.046) were estimated by all the patients in stage T3 with at least one positive node (*n* = 41,635).

**Table 2:** Estimated parameters across different stages

Parameter	All	T1 and T2	T3
*α*_*T*_	1.130	1.960	1.117
*β*_*T*_	3.201	10.453	2.957

**Fig. 2:**
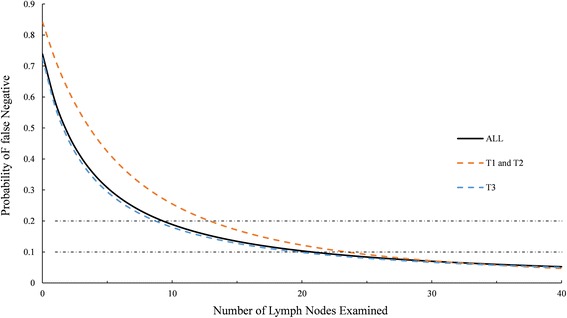
Probability of a false-negative as a function of number of nodes examined in a colon cancer patient with truly node-positive disease

The set of parameters was then used to estimate the probability of false-negative disease as a function of the number of examined nodes and tumor stages, which is different from studies of Joseph et al. [13] and Gönen et al. [12]. In stages T1 and T2, the probability of a false-negative node dissection was estimated at 72%, 54%, 26%, 12% and less than 10% for 1, 3, 10, 20 and greater than 26 nodes examined, respectively (Fig. 2 and Additional file 1: Table S1 in the supporting information). In stage T3, the probability of a false-negative node dissection was estimated at 57%, 39%, 18% and less than 10% for 1, 3, 10 and greater than 20 nodes examined, respectively. It is shown in Fig. 2 that the overall *α* and *β* were prone to underestimate the probability of false-negative in stages T1 and T2 and overestimate the probability of stage T3. The differences among probability of false-negative in three stages are less than 3% when more than 20 nodes are examined.

The observed prevalence of nodal disease is 38.1%, but accounting for false-negative patients, the corrected prevalence is 45.4% (Table 3). Underestimation of prevalence due to the existence of false- negatives is observed for all T stages, but its extent increases by T stage. As many as 57.0% of T3 colon cancer patients are estimated to have nodal disease, up from an observed rate of 48.5%.

**Table 3:** Observed and Corrected Prevalence

Prevalence(%)	All	T1	T2	T3
OP	38.1	11.1	19.3	48.5
CP	45.4	15.3	25.0	57.0

**Fig. 3:**
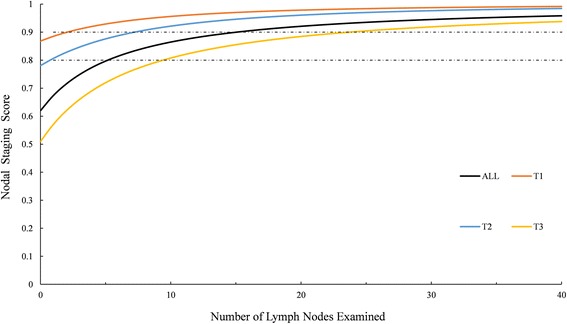
Nodal staging scores as a function of number of nodes examined in a colon cancer patient

Nodal staging scores were presented in Fig. 3 and Additional file 1: Table S2 in the supporting information. Patients with stage T1 and T2 will have more than a 90% chance of a correct pathologic diagnosis with three and eight examined nodes, respectively. The same level of accuracy requires twenty-four examined nodes in T3 patients. To achieve an 80% chance of a correct pathologic diagnosis, one, one and ten nodes are required to be examined for T1, T2 and T3 patients, respectively.

## Discussion

Adequate examined nodes are required for proper staging of colon cancer, and the number of LNs examined is associated with colon cancer survival [15]. When patients have too few nodes examined, clinicians face challenging decisions on under-staging because there would be a chance that this patient can be incorrectly treated as false-negative. By maximizing the prognostic discrimination between the grouped patients, many studies have sought a threshold for the minimum number of examined nodes [26–29], in which most of these suggestions have been made with regard to the number of examined nodes needed to accurately determine that a patient has occult node-negative cancer.

Recent studies subjected nodal staging to the statistical model by computing the false-negative rate and calculating the negative predictive value to define NSS that characterizes the adequacy of node-negative classification [12, 22, 30, 31]. However, most of these studies lose sight of the effect of tumor stage on the false-positive rate in the surgery of colon cancer. To the best of our knowledge, this study is the first to formulate the false-positive rate of occult nodal disease as a function of the number of examined nodes together with the T stage, and find a significant difference of false-positive rate among different T stages. Combining the number of examined nodes with the T stage, our approach established an individualized prognostication of the true nodal stage. Our results suggested an evident higher false-positive rate of T1 and T2 patients comparing to that of T3 patients when the number of examined nodes is less than 15, and a small but statistically significant higher false-positive rate of 3% when the number of examined nodes is between 15 and 20.

In addition, in order to minimize the bias caused by important confounders, we restricted our study population to first primary colon patients without neoadjuvent therapies. To facilitate the planning of the optimal individual treatment, we also evaluated whether other patient variables, such as patient sex and age, could lead to different false-positive rates. However, current data do not support that either patient sex or age can result in significantly different false-positive rates. Although we found that not all clinicopathological features are highly correlated with the false-positive rate in colon cancer, whether these features influence the false-positive rates in other categories of cancer are still open questions.

As a convenient tool to evaluate whether a node-negative colon cancer patient is adequately staged, a higher value of the calculated NSS implies a greater likelihood in the node-negative status of the patient for each tumor stage. Because the NSS calculates the probability of occult nodal disease as a function of the number of examined nodes and the T stage, this tool might give an estimation of the likelihood of node-metastasis more accurately than a simple cutoff of the number of examined nodes, and help clinicians judge the adequacy of nodal staging. Current guidelines recommended that at least 12 nodes needed to be examined as a quality indicator, based on a series of studies correlating the number of examined LNs with progression or survival [15–17]. However, we found that the number of nodes needed to be removed varies largely among patients according to different T stages [32]. For example, insisting on 12 nodes for patients with stages T1 and T2 seems unjustified, because the examination of 3 nodes for a T1 patient maintains the same level of confidence 90% with that of the examination of 8 nodes for a T2 patient. Consequently, our findings encourage the development of techniques to improve LNs harvest in color cancer especially for T3 patients.

Given the retrospective nature and a few key assumptions required for the calculation of NSS, there are several limitations of this study that warrant mention. First, although the assumptions on no false-positives and beta-binomial model are conservative and reasonable [12], the assumption that all nodes within a patient have the same probability of being involved is unlikely to hold in practice. We recognize, however, that the absence of the position of the examined nodes limit the justification on this assumption. The location of the examined nodes is substantial because nodes from an area of low likelihood of cancer may be less valuable than the nodes which are more likely to be involved with malignancy [31]. Consequently, prospective validation on this key assumption is required in the statistical model to estimate NSS in future. Secondly, the data from nodes-positive patients were used to interpret the data for the nodes-negative patients. We applied a bootstrap method to generate nodes-negative patients from observed nodes-positive patients by reducing one node that with equal possibility to be selected. The estimates of the false-positive rate from the bootstrap samples are in line with the estimates obtained only from nodes-positive patients, which justifies the rationality of the extension. Finally, as mentioned by many studies, the externally validation of the use of the NSS relies on the result of recurrence or death, to ensure that NSS can distinguish patients who are at high risk of having omitted occult nodal disease [12].

In conclusion, our study has several key distinctions. Strengths of our analysis included its novel application of tumor-stage-based false-positive rates into the calculation of NSS. The formula of prevalence and NSS varies in a way from the equations described in previous research. Our results allow clinicians to better understand the likelihood of missing nodal disease and assist the planning of optimal therapies.

## Conclusions

In conclusion, this study found that the false-negative rate of the examined lymph nodes in the colon cancer surgery depends preoperatively on the clinical T stage. A more accurate nodal staging score was developed to recommend a threshold on the minimum number of examined nodes regarding to the favored level of confidence for each T stage.

## Additional file

Additional file 1: Table S1. Probability of missing nodal disease (false negative, %) for selected values of the number of nodes examined. **Table S2.** Nodal staging score for selected values of the number of nodes examined. (DOCX 54 kb)

## Abbreviations

CP: corrected prevalence
LN: lymph node
NSS: nodal staging score
OP: observed prevalence
SEER: surveillance, epidemiology and end results
