# Supplementary material for: Assessing the adequacy of lymph node yield for different tumor stages of colon cancer by nodal staging scores
Source: BMC Cancer. 2017 Jul 25;17:498. doi: 10.1186/s12885-017-3491-2 (PMC5526283; doi:10.1186/s12885-017-3491-2)
Supplement: Additional file 1: Table S1. — Probability of missing nodal disease (false negative, %) for selected values of the number of nodes examined. Table S2. Nodal staging score for selected values of the number of nodes examined. (DOCX 54 kb) [file 12885_2017_3491_MOESM1_ESM.docx]

Table S1 Probability of missing nodal disease (false negative, %) for selected values of the number of nodes examined

| Stage | No. of Nodes Examined (%) | | | | | | | |
| --- | --- | --- | --- | --- | --- | --- | --- | --- |
|  | 1 | 2 | 5 | 10 | 12 | 15 | 20 | 25 |
| T1 T2 | 71.9 | 62.1 | 42.4 | 25.5 | 21.5 | 17.1 | 12.2 | 9.2 |
| T3 | 56.6 | 46.2 | 29.4 | 18.0 | 15.5 | 12.8 | 9.8 | 8.0 |
| ALL | 58.2 | 47.8 | 30.7 | 18.9 | 16.3 | 13.4 | 10.4 | 8.4 |

Table S2 Nodal staging score for selected values of the number of nodes examined

| Stage | No. of Nodes Examined | | | | | | | | |
| --- | --- | --- | --- | --- | --- | --- | --- | --- | --- |
|  | 1 | 3 | 5 | 8 | 10 | 12 | 15 | 20 | 24 |
| T1 | 0.885 | 0.911 | 0.929 | 0.947 | 0.956 | 0.963 | 0.970 | 0.978 | 0.983 |
| T2 | 0.806 | 0.847 | 0.876 | 0.907 | 0.922 | 0.933 | 0.946 | 0.961 | 0.969 |
| T3 | 0.571 | 0.660 | 0.719 | 0.779 | 0.808 | 0.830 | 0.855 | 0.885 | 0.901 |
